# Development and Evaluation of Cross-Linked Alginate–Chitosan–Abscisic Acid Blend Gel

**DOI:** 10.3390/polym15153217

**Published:** 2023-07-28

**Authors:** Daniel Bustos, Luis Guzmán, Oscar Valdés, Marcelo Muñoz-Vera, Luis Morales-Quintana, Ricardo I. Castro

**Affiliations:** 1 Centro de Investigación de Estudios Avanzados del Maule (CIEAM), Vicerrectoría de Investigación y Postgrado, Universidad Católica del Maule, Talca 3460000, Chile; dbustos@ucm.cl (D.B.); ovaldes@ucm.cl (O.V.); 2 Laboratorio de Bioinformática y Química Computacional (LBQC), Escuela de Bioingeniería Médica, Facultad de Medicina, Universidad Católica del Maule, Talca 3460000, Chile; 3 Departamento de Bioquímica Clínica e Inmunohematología, Facultad de Ciencias de la Salud, Universidad de Talca, Avenida Lircay, s/n, Casilla 747–721, Talca 3460000, Chile; lguzman@utalca.cl; 4 Multidisciplinary Agroindustry Research Laboratory, Universidad Autónoma de Chile, Cinco Pte. N°1670, Talca 3467987, Chile; marcelomunoz132@gmail.com; 5 Multidisciplinary Agroindustry Research Laboratory, Instituto de Ciencias Biomédicas, Facultad de Ciencias de la Salud, Universidad Autónoma de Chile, Cinco Pte. N°1670, Talca 3467987, Chile; 6 Multidisciplinary Agroindustry Research Laboratory, Instituto de Ciencias Aplicadas, Facultad de Arquitectura, Construcción y Medio Ambiente, Universidad Autónoma de Chile, Cinco Pte. N°1670, Talca 3467987, Chile

**Keywords:** alginate–chitosan blend, abscisic acid, complex, ionic interaction, calcium cross-linking

## Abstract

Abscisic acid (ABA) has been proposed to play a significant role in the ripening of nonclimacteric fruit, stomatal opening, and response to abiotic stresses in plants, which can adversely affect crop growth and productivity. The biological effects of ABA are dependent on its concentration and signal transduction pathways. However, due to its susceptibility to the environment, it is essential to find a suitable biotechnological approach to coat ABA for its application. One promising approach is to utilize alginate and chitosan, two natural polysaccharides known for their strong affinity for water and their ability to act as coating agents. In this study, an alginate–chitosan blend was employed to develop an ABA cover. To achieve this, an alginate–chitosan–abscisic acid (ALG–CS–ABA) blend was prepared by forming ionic bonds or complexes with calcium ions, or through dual cross-linking. This was done by dripping a homogeneous solution of alginate–chitosan and ABA into a calcium chloride solution, resulting in the formation of the blend. By combining the unique properties of alginate, chitosan, and ABA, the resulting ALG–CS–ABA blend can potentially offer enhanced stability, controlled release, and improved protection of ABA. These characteristics make it a promising biotechnological approach for various applications, including the targeted delivery of ABA in agricultural practices or in the development of innovative plant-based products. Further evaluation and characterization of the ALG–CS–ABA blend will provide valuable insights into its potential applications in the fields of biomedicine, agriculture, and tissue engineering.

## 1. Introduction

Alginate and chitosan are biopolymer fibers derived from natural sources. Both alginate and chitosan possess unique properties such as biocompatibility, biodegradability, nontoxicity, and low cost, making them widely used in various industries such as food, pharmaceuticals, biomedicine, and textiles. These materials possess excellent biocompatibility, biodegradability, nontoxicity, nonimmunogenicity and are cost-effective [1,2,3,4].

Alginate is a polysaccharide composed of alternating β-D-mannuronate (M) and α-L-guluronate (G) units [5,6]. The M and G monomeric structures of alginate can form a gel or polymeric mesh through ionic exchange with calcium ions. In an aqueous solution, the carboxylic groups of alginates interact with Ca^2+^ ions, resulting in the formation of “boxes” or “eggs” between GG and MM blocks [7,8].

Chitosan, on the other hand, is a weak cationic polysaccharide primarily composed of (1,4)-linked 2-amino-2-deoxy-β-D-glucan units [9]. Chitosan could form ionic bonds with structures containing carboxylic acids, such as alginates [10]. Additionally, its amino groups make it a pH-sensitive material, which is useful for drug delivery applications [11,12].

Blending polymers is a well-known and effective method for improving the performance of polymer materials. Chitosan and alginate can be blended due to the similarity in their carboxyl groups [13]. This blending allows the formation of systems through interpolyelectrolyte complex reactions, leading to properties such as release-retarding behavior, particularly when induced via alginate–chitosan complexation and calcium cross-linking [14,15].

This blend has been used in various applications, including the rapid adsorption of heavy metal ions in wastewater [16], the removal of Cr^6+^ in water treatment [17], the encapsulation of Vitamin B2 and β-Carotene in multilayer alginate/chitosan systems [18], the release of sodium ceftriaxone [19], and pesticide removal [10].

**Figure 1:**
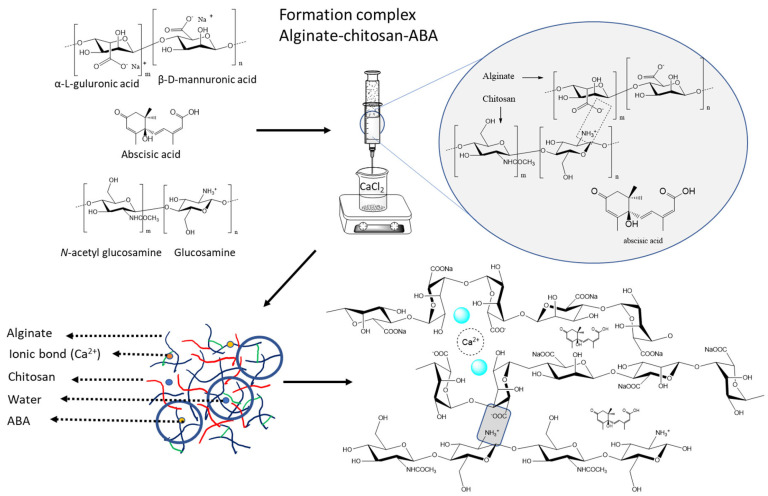
Schematic representation of the sequential formation of the alginate–chitosan–ABA (abscisic acid) complex with calcium cross-linking, step-by-step process involved in creating the complex.

On the other hand, abscisic acid (ABA) is a phytohormone that offers several advantages in agriculture and horticulture. It enhances abiotic stress tolerance, improves crop quality, regulates growth and development, and controls fruit ripening [20,21,22]. ABA helps plants withstand drought, salinity, and extreme temperatures, while promoting the synthesis of beneficial compounds [20]. Its application influences seed germination, shoot elongation, root development, and leaf senescence [23]. ABA also prolongs fruit shelf life by delaying ripening [22]. Careful application and adherence to regulations are important for maximizing benefits and minimizing potential drawbacks. Hence, ABA plays a role in triggering the production of secondary compounds in strawberries when exposed to salt and drought stresses. However, an excessive amount of ABA can lead to plant death and the formation of undersized fruits [22]. Therefore, it is crucial to administer ABA in a controlled manner, using optimal dosages, to achieve a maximal response without compromising plant vitality and the desirable sensory qualities of the fruits. For this, the present study focused on the development and evaluation of a blend gel composed of cross-linked alginate, chitosan, and abscisic acid and determining if it is possible to build a gel blend that captures ABA (Figure 1).

The goal is to assess the properties and potential applications of this unique gel formulation. Alginate and chitosan, both biocompatible polymers, are cross-linked to enhance the gel’s stability and mechanical strength. The addition of abscisic acid, a plant hormone known for its diverse biological functions, provides additional functionalities to the gel. Through comprehensive evaluation, including characterization of physical properties, release kinetics, and biological activity, it acts as a regulator in stress adaptation and growth modulation, controlling seed dormancy and germination. ABA facilitates plant survival during unfavorable conditions and promotes stomatal closure to reduce water loss through transpiration [24,25].

This study aimed to understand the potential of the cross-linked alginate–chitosan–abscisic acid blend gel for various applications in fields such as biomedicine, agriculture, and tissue engineering.

## 2. Materials and Methods

### 2.1. Building the Molecular Structures of the Polymers

The three-dimensional structures of β-D-mannopyranuronate (M) and α-L-gulopyranuronate (G) were obtained based on previous work conducted in our laboratory [26]. Similarly, the structure of abscisic acid (ABA) was obtained from a protein structure co-crystallized with ABA (PDB code: 3JRQ), and the chitosan polymer structure was obtained from a study by Valdes et al. (2021) [27]. The pKa values for M (approximately 3.4) and G (approximately 3.6) were obtained from Bustos et al. (2022) [26], while the pKa value for ABA was evaluated using the Epik software from the Maestro-Schrödinger program [28].

To generate the molecular systems, both alginate and chitosan polymers were used according to the methodology described by Valdes et al. (2021) [27]. The alginate:chitosan ratio was varied to create three different systems (1:1, 1:2, and 2:1). In these systems, the alginate and chitosan blocks were formed using G8-block/M8-block and GM8-block structures, respectively, with chains of chitosan consisting of 16 monomers (Figure 1). The polymer chains were randomly distributed within a sphere with a radius of 60 Å using PACKMOL software v.16.070.0 [29]. Finally, 20 ABA molecules were randomly placed in a smaller sphere with a radius of 50 Å.

### 2.2. Molecular Dynamics Simulations of Alginate/Chitosan with and without ABA

For the construction of each of the three different systems (1:1, 1:2, and 2:1 alginate:chitosan ratios), they underwent energy minimization using the same conditions as described by Bustos et al. (2022) [26]. The systems were simulated using the Desmond/Maestro-Schrödinger suite [28], and the OPLS v.2005 force field [30] was applied. The default relaxation protocol was employed, consisting of five short simulations, following the protocols outlined in Bustos et al. (2022) [26]. The production simulations were performed in an NPT ensemble at ambient conditions (pressure = 1 atm and temperature = 300 K), with a duration of 100 ns for each system, performed in triplicate. After evaluating the interaction behavior of alginate/chitosan in the three systems, three additional simulations were conducted for each system, this time including the 20 ABA molecules.

Chitosan with a molecular weight (160 kDa) was purchased from Sigma-Aldrich. Sodium alginate (medium viscosity) was purchased from Sigma. The ABA used (+/−)-abscisic acid mixed (sis-trans) isomers (phytotechlab). Milli-Q water with a resistivity of 18.2 MΩ*cm was used, and calcium chloride hexahydrate (Sigma Aldrich, St. Louis, MI, USA, 98%) was employed as well. All other chemicals used were of reagent grade.

### 2.3. Preparation of the Alginate–Chitosan–ABA Complex with Calcium Cross-Linking

To prepare the sodium alginate and chitosan blend solutions, the procedure hereafter was followed. First, 1.5 g of each polymer were weighed and dissolved in 100 mL of Milli-Q water 1.5% (*w*/*v*). The dissolution process was carried out at room temperature while maintaining continuous mechanical stirring overnight. This extended duration ensured thorough mixing and complete hydration of the polymers, resulting in homogeneous blend solutions. Then, the different complexes (1 mL) were formed between chitosan, alginate, and abscisic acid at alginate:chitosan ratios of 2:1, 1:1, and 1:2.

Additionally, 15 mg of ABA were weighed and dissolved in 10 mL of Milli-Q water. Then, 1 mL of the abscisic acid (ABA) was incorporated into the different blend solutions of alginate and chitosan. This addition of ABA provided the desired bioactive component within the blend system, imparting specific functional properties to the resulting complex.

The blend solutions were transferred into a 20 mL injection needle of 18 G (outer diameter = 1.27 mm) and added dropwise into a 2% (*w*/*v*) calcium chloride solution. Spherical beads were formed through mechanical stirring for 15 min and subsequently washed with Milli-Q water before being dried at room temperature.

### 2.4. Preparation of Samples and Thermogravimetric Analysis

The water content of the samples was determined using thermogravimetric analysis (TGA) by measuring the weight loss in the temperature range of 50 to 200 °C.

To assess the stability of the complex, dried samples obtained from a pressure chamber were subjected to lyophilization using a Biobase BK-FD10P, China, freeze dryer. The thermogravimetric analysis (TGA) was performed on all samples using 5 mg of each sample to evaluate the chemical characteristics of the degradation process. The TGA measurements were conducted using an STD 650 (TA Instruments Thermal Analyzer, New Castle, DE, USA). Each sample was heated at a constant rate of 10 °C/min in the presence of air as the reactive gas with a mass flow of 50 mL/min. Additionally, a protective gas of N_2_ at a flow rate of 50 mL/min was used in the electronic balance.

### 2.5. Attenuated Total Reflection–Fourier Transform Infrared (ATR–FTIR) Spectroscopy

All samples were carefully prepared and subjected to analysis using FTIR spectroscopy (Cary-360) (Agilent Scientific Instruments, Santa Clara, CA, USA), equipped with an attenuated total reflection (ATR) module, ensuring precise and accurate results. FTIR spectroscopy is a powerful technique that provides valuable information about the molecular structure and chemical composition of substances. The absorbance measurements were performed in the range of 500 to 4000 cm^−1^, with a resolution of 4 cm^−1^.

### 2.6. Determination of Abscisic Acid (ABA)

To determine the content of abscisic acid (ABA), flasks containing 0.9 mg of the sample in 4 mL of Milli-Q water were sonicated in an ultrasonic bath for 1 h at a frequency of 50 kHz and a power of 100 W. After sonication, an aliquot of the supernatant was taken for analysis. The amount of ABA was determined spectrophotometrically using a calibration curve. Solutions with concentrations ranging from 0.1 to 0.5 mg/mL of ABA were prepared, and the samples were measured at 265 nm [31] using a spectrophotometer (Thermo Spectronic, Genesys 10 UV, Waltham, MA, USA). The recorded absorbance values were compared against the calibration curve to calculate the content of ABA in the samples. The concentration of ABA in the complex was expressed as mg of ABA per mg of complex.

## 3. Results and Discussion

### 3.1. In Silico Nanoparticle Formation

**Figure 2:**
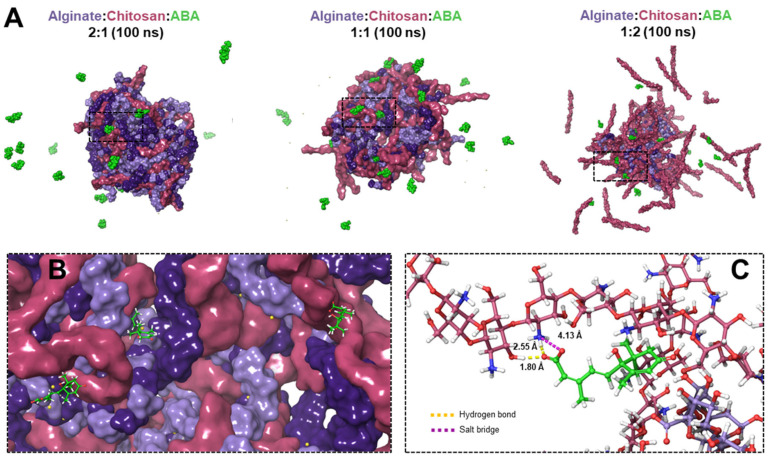
Alginate–chitosan nanoparticle formation: (**A**) The final time step (100 ns) for each complex is depicted, where both types of alginate chains are colored in lilac and purple. Meanwhile, chitosan chains and ABA molecules are in fuchsia and green colors, respectively. (**B**) Approach to the intermolecular cavities of the nanoparticle where the ABA molecules are located and (**C**) intermolecular interactions between ABA and alginate:chitosan chains.

**Figure 3:**
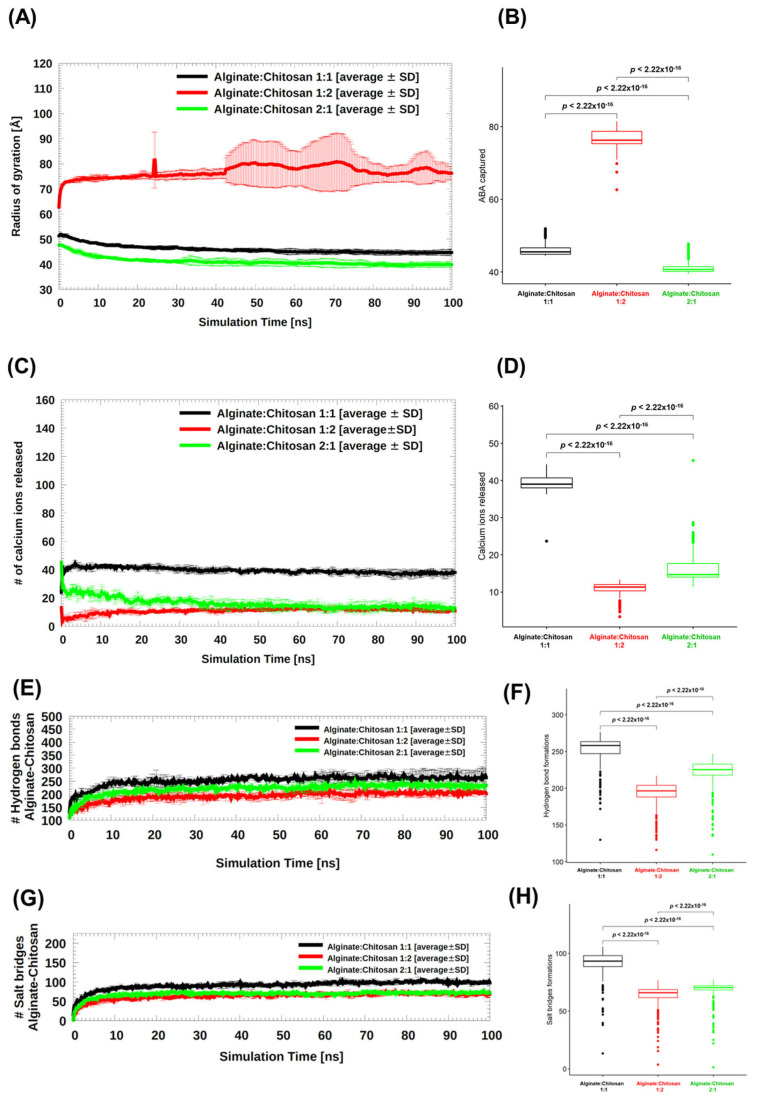
Stabilization of the nanoparticle over the simulation time: through (**A**) radius of gyration, (**C**) the number of calcium ions released, and intermolecular interactions between alginate and chitosan: (**E**) hydrogen bonds and (**G**) salt bridges. Statistical analysis for each molecular descriptor previously mentioned is shown in (**B**,**D**,**F**,**H**).

Molecular dynamics (MD) simulation studies were conducted to investigate the interaction between chitosan and alginate in an aqueous environment and evaluate the stability of the formed nanostructures. The stability of calcium ions (Ca^2+^) in the three different systems with varying alginate:chitosan ratios (1:1, 1:2, and 2:1) was analyzed by monitoring the radius of gyration (Rg) and the number of released Ca^2+^ ions during the simulations (Figure 2). According to the MD simulations, the most stable complexes were observed in the 2:1 alginate:chitosan ratio system (Figure 3A,B, represented by green color). In contrast, the system with twice the amount of chitosan molecules compared to alginate (1:2 alginate:chitosan system) did not form a compact particle after 100 ns of simulation (Figure 2). Additionally, the interaction between ABA molecules and the different complexes has been studied through bioinformatic analysis (see Figure 2B,C) and showed that there are salt bridge interactions between the carboxylic and hydroxylic groups of abscisic acid with amine groups from chitosan; there are also hydrogen bond interactions between the oxygen of the carboxylic group of the acid and the hydroxylic group of the polymers of the complex (see Figure 2B,C).

The release of Ca^2+^ ions in the three systems is depicted in Figure 3C, and the statistical analysis is presented in Figure 3D. Initially, each system contained 160 Ca^2+^ ions. The 1:1 alginate/chitosan system showed the highest number of released Ca^2+^ ions, indicating that it required less cross-linking to stabilize the nanoparticle, resulting in the rapid release of excess calcium ions (Figure 3C,D, represented by black color). This result was expected, since the 1:1 system requires a lower amount of Ca^2+^ compared to the 2:1 system (alginate:chitosan), as the latter contains twice the number of negatively charged alginate chains (with −1 charge per monomer) compared to chitosan chains (with +1 charge per monomer). The higher the number of negative charges, the more calcium ions are needed to stabilize the system.

Although the high amount of chitosan chains in the 1:2 system (Figure 3C,D, represented in red color), and consequently the high availability of positive countercharges to stabilize the alginate chains, this system systematically shows less loss of calcium ions compared to the other systems, showing that even in the high presence of chitosan, calcium ions are thermodynamically more stable as cross-linkers. The electrostatic repulsions exerted in this system end up expelling a large number of chitosan chains (Figure 2A). After approximately 40 ns of simulation, both systems (1:2 and 2:1, represented in red and green respectively), reach the same number of ions inside the nanoparticle.

The statistical comparison among the three systems demonstrated a significant difference, including the comparison between 1:2 and 2:1, which appeared quite similar throughout the simulation trajectory (Figure 3D).

Concerning the hydrogen bond (HB) and salt bridge (SB), Figure 3E,G showed the interactions between the alginate and chitosan chains in the three systems. The same trend can be seen for both types of interactions, with the 1:1 system generating the most interactions and the 1:2 system generating the fewest interactions (Figure 3E,G). When comparing the formation of HB against SB, it is observed that there are between two and five times more HB than SB in all systems studied (Figure 3E,G). It is reasonable that the 1:2 system generates the least interactions between the alginate and chitosan chains due to the partial compaction seen in this system. For both HB and SB, there are statistical differences between the three pairs of systems to be compared: 1:1 vs. 1:2, 1:1 vs. 2:1, and 1:2 vs. 2:1 (Figure 3F,H).

### 3.2. Characterization of Complex, Hydration, and Size

The hydration capacity of alginate–chitosan complexes was assessed through thermogravimetric analysis (TGA), and its correlation with sample size was investigated. The TGA curves revealed that the hydration capacity varied depending on the ratio of alginate to chitosan in the complex. Table 1 presents the results, indicating differences in the amounts of weakly and strongly bound water within the complex, particularly in the temperature range of 50 to 180 °C [7,32].

**Figure 4:**
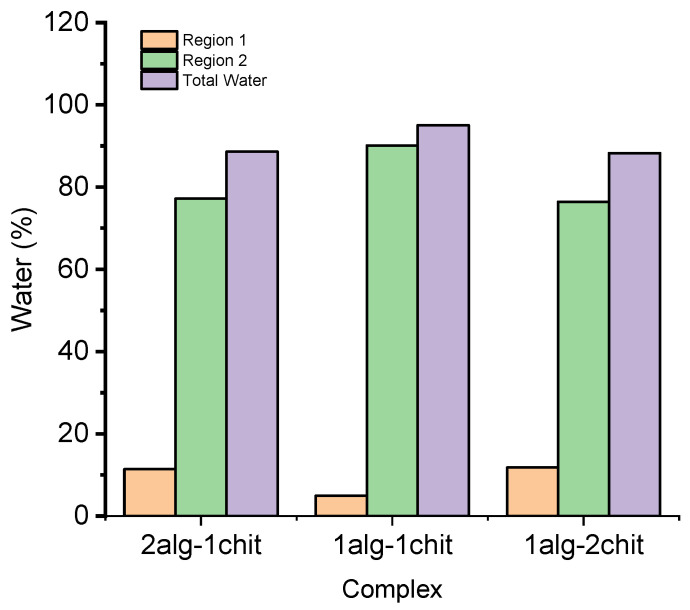
Water contents determined through TGA analysis.

The analysis on the absorption of water in the alginate–chitosan complexes formed is primarily attributed to the coordination of calcium ions with water molecules and oxygen atoms, from the carboxyl chains of alginate [33]. This intricate interaction significantly influences the complex’s water absorption properties and has been extensively studied for its importance in various applications. In the alginate–chitosan complexes, calcium ions play a pivotal role in facilitating water absorption. The presence of calcium ions leads to the cross-linking of the alginate chains, forming a three-dimensional network structure. This network structure provides numerous sites for water molecules to interact and become trapped within the complex [7]. Table 1 also demonstrates that a higher concentration of chitosan in the complex results in greater water absorption capacity due to fewer interactions within the chains, creating more interstitial spaces and fewer calcium ions that displace water molecules. This observation may be associated with the lower concentration of free water within the complex (see Figure 4).

Furthermore, the structure of chitosan and alginate suggests that water molecules can be bound by three polar groups: amine, carboxyl, and hydroxyl, present in chitosan, sodium alginate, and the structure of both polymers, respectively [34]. Additionally, the size of the complex is related to the ratio of alginate to chitosan.

**Figure 5:**
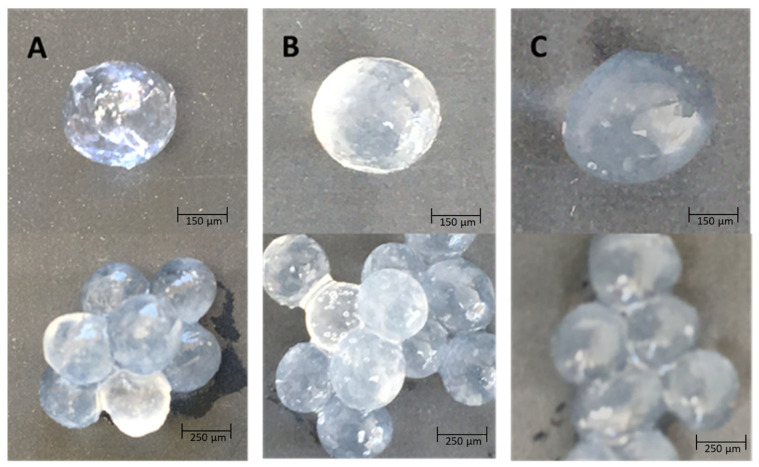
Cross-linked alginate–chitosan–abscisic acid blend gel for delivery system; alginate:chitosan ratios of (**A**) 2:1, (**B**) 1:1, and (**C**) 1:2.

The results indicate that ABA molecules within the different complexes interact with the functional groups of the polymers, leading to the displacement of calcium ions and potentially causing the deformation of the complex spheres (refer to Figure 5) [35]. In the case of alginate–chitosan complexes with ABA, this deformation occurs due to ABA molecules interacting with the carboxyl and amine groups of the polymers, thereby reducing the ionic interactions between the polymers. These interactions between ABA and the polymer groups result in the modulation of the ionic interactions between the polymers, leading to structural changes within the complex.

### 3.3. Thermogravimetric Analysis (TGA) and (DTG) Curves of the Complex Formed in This Study

**Figure 6:**
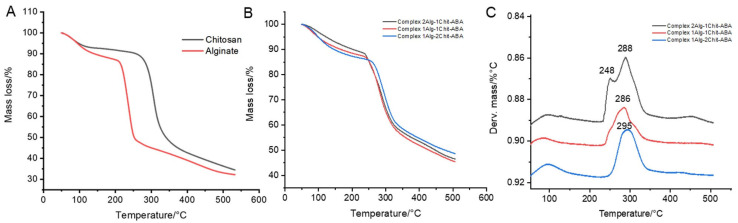
Thermogravimetric analysis of the different complex. (**A**) thermogravimetric analysis (TGA) for the polymers chitosan and alginate; (**B**) thermogravimetric analysis for complex alginate:chitosan (different ratio); (**C**) DTG for complex alginate:chitosan (different ratio).

The results from experimental data provided empirical evidence supporting the gelation stability of calcium ions and the interaction between alginate fibers and chitosan, mainly, an ionic bond between three polar groups: amine, carboxyl, and hydroxyl. The TGA analysis indicated that the most stable complex ratio was alginate:chitosan 2:1, while the alginate:chitosan 1:2 ratio exhibited lower stability (refer to Figure 6A).

Furthermore, the ABA compounds can be encapsulated within the complex structure through ionic interactions, primarily with the amine and carboxylate groups (in this study, ABA, and chitosan).

The spectra depicted in Figure 5 show the distinct structures and thermal stability of alginate and chitosan, both of which exhibit different degradation onset temperatures. Alginate degrades at approximately 200 °C, whereas chitosan degrades at around 250 °C [36]. This difference in degradation behavior can be attributed to the variation in polymer structure by the interaction between alginate is a linear copolymer composed of β-D-mannuronic acid and α-L-guluronic acid, with chitosan the which is a linear polymer composed of β-(1→4)-linked D-glucosamine and N-acetyl-D-glucosamine units [37]. However, the presence of transition metal ions or salts enhances the stability of both structures [38,39].

The TGA analysis of cross-linked alginate–chitosan demonstrated an increase in stability compared to alginate alone [40]. The cross-linking between alginate and chitosan, facilitated by the interaction with cross-linking agents such as CaCl_2_, contributed to higher thermal stability [41]. The weight loss of the cross-linked complex was found to be less than 10% up to 300 °C.

### 3.4. Characterization of Complex by Attenuated Total Reflection–Fourier Transform Infrared (ATR–FTIR) Spectroscopy

ATR–FTIR spectroscopy was employed to characterize the complex and analyze its formation, in addition to providing information about the functional groups present. The spectra of all the complexes were compared and analyzed, revealing that abscisic acids, alginate, chitosan, and calcium can form stable complexes capable of effectively integrating abscisic acids’ molecules within their interstitial spaces.

**Figure 7:**
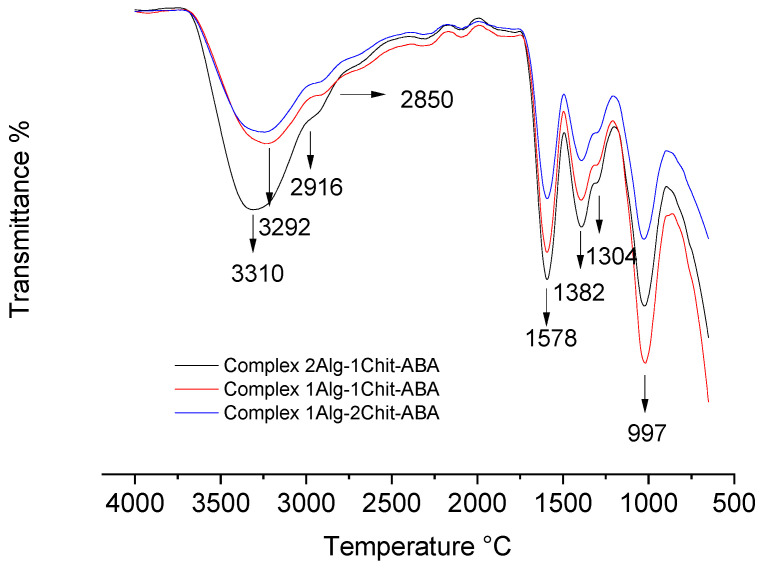
Attenuated Total Reflection–Fourier Transform Infrared (ATR–FTIR) spectroscopy of the different complexes formed between chitosan, alginate, and abscisic acid.

The FTIR spectrum (Figure 7) can offer insights into the functional groups present in the complex from the different polymeric structures. For instance, the hydroxyl group (OH) stretching of chitosan and alginate is observed at 3310 cm^−1^ [7]. Furthermore, the FTIR spectrum of alginate and chitosan shows peaks corresponding to the antisymmetric stretch vibration of the C-O-C bond at 997 cm^−1^ and the stretching vibration of the carboxylate ion COO- at 1578 cm^−1^ [42].

Additionally, the FTIR spectrum typically exhibits peaks related to the amino group (NH_2_) stretching vibration at 3292 cm^−1^ [43] and the C-O-C stretching vibration at 997 cm^−1^. However, there is an overlap between the N-H bending of the amino group in chitosan and the carboxylate ion vibration of alginate at 1578 cm^−1^ [34].

Finally, the pronounced peaks at 2916 cm^−1^ and 2850 cm^−1^ indicate the presence of asymmetric CH stretching and symmetric CH stretching, respectively, suggesting the presence of abscisic acids (ABA) molecules within the complexes (Figure 7).

## 4. Conclusions

In conclusion, the results obtained from the molecular dynamics (MD) simulations indicate that the 2:1 alginate:chitosan ratio system exhibited the most stable complexes, as evidenced by the compact particle formation and minimal release of calcium ions. On the other hand, the 1:2 alginate/chitosan system showed initial instability and leakage of calcium ions, which was later compensated for by the re-entry of ions into the system. The 1:1 alginate/chitosan system displayed the highest number of released calcium ions, suggesting a lower degree of cross-linking and greater need for stabilization. The analysis of hydrogen bond (HB) and salt bridge (SB) interactions further supported these findings, with the 1:1 system demonstrating the most interactions. The hydration capacity of the complexes varied with the alginate:chitosan ratio, with higher chitosan concentrations leading to greater water absorption due to increased interstitial spaces. Thermogravimetric analysis (TGA) confirmed the stability of the complexes, with the 2:1 ratio showing the highest stability. ATR–FTIR spectroscopy provided insights into the functional groups present in the complex, such as hydroxyl groups, C-O-C bonds, and amino groups. The presence of characteristic peaks corresponding to ABA molecules within the complexes suggests successful integration of ABA into the interstitial spaces. Overall, these findings contribute to our understanding of the formation, stability, and characterization of the chitosan–alginate complexes, which can have implications in various applications, including drug delivery systems.

## Figures and Tables

**Table 1:** Physical–chemical characteristics of the different complexes formed between chitosan, alginate, and abscisic acid.

Sample	Complex + ABA		
Size mm (n = 3)	0.320 ± 0.02	0.345 ± 0.04	0.410 ± 0.04
Water abs (%) (region 2)	77.22	76.44	90.12
Total water (%)	88.61	88.22	95.06
Ratio: alginate:chitosan	2:1	1:1	1:2
Concentration of ABA in the complex per 0.9 mg complex	0.038	0.023	0.007

## Data Availability

Not applicable.
